# Melatonin Attenuates H_2_O_2_-Induced Oxidative Stress by Restoring Redox Balance, Mitochondrial Integrity and Reducing Apoptosis in Buffalo Fibroblasts

**DOI:** 10.3390/antiox15040508

**Published:** 2026-04-20

**Authors:** Priya Dahiya, Manu Mangal, Srishti Bhatia, Neha Sharma, Ashish Sindhu, Bhavya Maggo, Meeti Punetha, Renu Bala, Pradeep Kumar, Dharmendra Kumar

**Affiliations:** Animal Physiology and Reproduction Division, ICAR-Central Institute for Research on Buffaloes, Sirsa Road, Hisar 125001, India; priyadahiya3154@gmail.com (P.D.); nehavashist000@gmail.com (N.S.); ashishsindhu2920@gmail.com (A.S.);

**Keywords:** buffalo fibroblasts, reactive oxygen species, melatonin, mitochondrial membrane potential, apoptosis

## Abstract

Oxidative stress critically affects cellular viability and function under in vitro culture conditions, often compromising physiological integrity of somatic cells used in livestock biotechnology. This study aimed to investigate hydrogen peroxide (H_2_O_2_)-induced oxidative stress in buffalo fibroblasts and evaluated the cytoprotective effects of melatonin, focusing on redox homeostasis, mitochondrial function, apoptosis, and antioxidant defence. Fibroblasts were exposed to graded concentrations of H_2_O_2_ (100–1000 µM) for 2 h, followed by treatment for 72 h in culture media with and without melatonin (10^−9^ M). Oxidative stress markers, including GSSG/GSH ratio, ROS generation, mitochondrial membrane potential (MMP), and apoptosis, were assessed using flow cytometry and biochemical assays, while antioxidant (GPx, SOD, CAT) and apoptotic (BAX, Caspase 9) gene expression was analyzed by qPCR. H_2_O_2_ exposure induced a dose-dependent increase in oxidative stress, evidenced by elevated ROS, redox imbalance, mitochondrial depolarization, and enhanced apoptosis. Severe oxidative damage was observed at higher H_2_O_2_ (500–1000 µM) concentrations. Melatonin (MT) significantly (*p* ≤ 0.05) alleviated oxidative stress under mild to moderate conditions (100–200 µM H_2_O_2_) by restoring redox homeostasis, preserving mitochondrial integrity, suppressing ROS accumulation, enhancing antioxidant defence, and reducing apoptosis. However, its protective efficacy was lost under severe oxidative stress, indicating a defined redox threshold beyond which cellular damage becomes irreversible. These findings suggest that melatonin exerts cytoprotective effect against oxidative stress within a limited oxidative window and provide mechanistic insights for improving fibroblasts culture systems in livestock biotechnology and regenerative applications.

## 1. Introduction

Oxidative stress is a critical determinant of cellular homeostasis and viability. It occurs when the generation of reactive oxygen species (ROS) surpasses the ability of cellular antioxidant defence systems to neutralize them. Under physiological conditions, minimal concentrations of ROS, such as superoxide anion and hydrogen peroxide (H_2_O_2_), function as vital signalling molecules that govern proliferation, differentiation, and metabolic adaptation. However, excessive or persistent accumulation of ROS induces oxidative damage to lipids, proteins, and nucleic acids, ultimately causing mitochondrial dysfunction, apoptosis, and cellular senescence [1,2].

In vitro culturing of fibroblasts is extensively employed to study cellular responses to external stimuli, assess drug toxicity, facilitate somatic cell nuclear transfer (SCNT), perform genome editing, enable cryopreservation of genetic resources, and investigate fundamental biological processes such as differentiation and disease progression. Fibroblasts represent one of the most extensively employed somatic cell types in livestock biotechnology due to their ease of isolation, genetic stability, robust proliferative capacity, and appropriateness for sustained culture maintenance [3,4,5]. However, oxidative stress remains a critical factor influencing cellular function during in vitro culture. Excessive accumulation of ROS disrupts redox homeostasis, impairs mitochondrial function, and alters overall cellular physiology, thereby affecting cellular behaviour and experimental outcomes [6,7,8]. Among ROS, H_2_O_2_ is a relatively stable, membrane-permeable molecule that plays a dual role in cellular physiology and pathology. At low concentrations, H_2_O_2_ serves as a mediator of redox signalling, while at high levels it promotes oxidative stress, disturbs redox homeostasis, and initiates mitochondrial dysfunction and apoptosis [9,10]. Due to these characteristics, H_2_O_2_ is extensively utilized as an experimental inducer of regulated oxidative stress in vitro. Excessive exposure to H_2_O_2_ depletes intracellular antioxidant defences, notably reduced glutathione (GSH), elevates levels of oxidized glutathione (GSSG), and induces oxidative damage in a dose- and time-dependent manner [11].

Melatonin (N-acetyl-5-methoxytryptamine) is a highly conserved indoleamine that exhibits significant antioxidant and cytoprotective properties [12]. Beyond its conventional role in regulating circadian rhythms, melatonin functions as a direct scavenger of ROS and reactive nitrogen species, as well as an indirect antioxidant by promoting glutathione synthesis and enhancing the activity of antioxidant enzymes such as superoxide dismutase, glutathione peroxidase, and catalase [13,14]. Particularly, melatonin accumulates within mitochondria, where it stabilizes mitochondrial membranes, enhances the efficacy of electron transport, decreases electron leakage, and inhibits the production of mitochondrial ROS [15,16]. Melatonin additionally promotes cell viability and maintains redox homeostasis across various in vitro systems, including embryonic cells, stem cells, and somatic cells subjected to oxidative or cryogenic stress [17,18]. In livestock systems, melatonin supplementation has been shown to enhance antioxidant capacity, mitochondrial function, and developmental competence under stress conditions [19,20,21]. However, recent research suggests that the cytoprotective effectiveness of melatonin is highly dependent on dosage and is limited by the extent of oxidative stress. Beyond a critical oxidative threshold, antioxidant defences become insufficient to reestablish cellular homeostasis, resulting in irreversible mitochondrial damage and the initiation of apoptosis [22,23].

Melatonin has antioxidant potential, but evaluation of its protective effects against H_2_O_2_-induced oxidative stress in buffalo fibroblasts is limited, particularly in mitochondrial function, glutathione redox homeostasis, apoptosis, and gene transcription. Optimization of fibroblast culture conditions and their use in bovine biotechnology and regenerative medicine require a mechanistic understanding of these processes. This study reveals how melatonin protects buffalo fibroblasts from H_2_O_2_-induced oxidative injury by restoring redox equilibrium, preserving mitochondrial function, and reducing apoptotic signalling. Our findings provide a quantitative and functional framework for redox regulation in buffalo somatic cells by establishing the oxidative stress threshold beyond which cellular homeostasis fails and the effective concentration window of melatonin for protection. This study fills a gap in large-animal redox biology and shows melatonin as a powerful redox regulator that boosts fibroblast resilience under in vitro stress. These findings address buffalo fibroblast culture stability, genome editing, cryopreservation, and regenerative and reproductive biotechnology.

## 2. Materials and Methods

All reagents and culture media employed in the investigation were procured from Sigma-Aldrich (St. Louis, MI, USA) unless stated otherwise. Fetal bovine serum was purchased from Gibco (Grand Island, NY, USA), and all disposable culture ware was obtained from Nunc (Roskilde, Denmark). Cells were maintained under standard culture conditions at 37 °C in a humidified incubator with 5% CO_2_. H_2_O_2_ stock solution (1 M) was prepared in distilled water and subsequently diluted to the desired concentrations for treatment in culture medium. Melatonin (Cat. No. M5250-5G; Sigma-Aldrich, St. Louis, MI, USA) was obtained as a crystalline powder, dissolved in dimethyl sulfoxide (DMSO) to prepare a 1 M stock solution, and stored at −20 °C until use.

### 2.1. Experimental Design

Buffalo fibroblasts were exposed to increasing concentrations of H_2_O_2_ (0, 100, 200, 500, and 1000 µM) for 2 h to induce acute oxidative stress. To examine the immediate cellular response, one subset of cells was collected immediately after exposure (H_2_O_2_ × 2 h group). To evaluate post-stress recovery, another subset was cultured for an additional 72 h in fresh medium without melatonin (H_2_O_2_ without MT group). To assess the cytoprotective potential of melatonin, a parallel subset of H_2_O_2_-treated cells was cultured for 72 h in the presence of melatonin (10^−9^ M) (H_2_O_2_ with MT group). The melatonin dose was selected based on previous reports [20,24], as low concentrations promote cell proliferation, whereas higher doses exert cytotoxic effects, likely due to their pro-oxidant activity leading to ROS generation, DNA damage, cell cycle arrest, and apoptosis [25]. Untreated cells maintained under identical conditions served as the control. Accordingly, cells were categorized into the following experimental groups described in Table 1.

### 2.2. Induction of Oxidative Stress and Melatonin Supplementation in Buffalo Fibroblasts

Fibroblasts were derived from skin biopsies collected from the tail region above the anus and confirmed using cell-type-specific markers such as vimentin, cytokeratin-18, and tubulin (Supplementary File, Figure S1). Cells were cryopreserved at passages 2 and 3 in medium supplemented with 10% DMSO using a slow-freezing protocol [3]. Fibroblasts at passage 2 were washed twice at 150 g with culture media containing DMEM/F12 medium (#D8437) containing 10% fetal bovine serum (#F2442) and 1% antibiotic-antimycotic solution (#A5955). Viability was assessed using trypan blue exclusion, and cells were plated at 1.5 × 10^4^ cells per well in 24-well plates with 1 mL culture media and incubated for 24 h. The medium was then replaced with fresh medium containing different concentrations of H_2_O_2_ (0, 100, 200, 500, and 1000 μM) and cultured for 2 h. After treatment with H_2_O_2_, cells were cultured in medium with and without melatonin (10^−9^ M) for 72 h. Cells were then processed for RNA isolation and evaluation of mitochondrial membrane potential (MMP), ROS generation and apoptosis using appropriate dye.

### 2.3. Evaluation of Intracellular Redox Status via GSSG/GSH Assay

Following treatment in groups, cells were harvested, washed with ice-cold phosphate-buffered saline (PBS), and lysed using the cell lysis buffer. Cell lysates were centrifuged to remove insoluble debris, and supernatants were collected for biochemical analyses. Intracellular redox status was then assessed by measuring reduced (GSH) and oxidized (GSSG) glutathione levels using a fluorometric GSH/GSSG detection assay kit (#AB138881, Cambridge, UK, Abcam) according to the manufacturer’s instructions. Briefly, samples were deproteinized, neutralized, and incubated with thiol-reactive fluorescent probes. Flat bottom 96 well plate was prepared with the standards, and test wells were prepared by mixing the supernatant with two reagents provided in the test kit. The plate was then incubated at 37 °C for 40 min. Fluorescence was measured using a microplate reader (Biotek Epoch microplate spectrophotometer, Agilent Technologies, Santa Clara, CA, USA) at an excitation/emission wavelength of 490/520 nm. GSH and total glutathione concentrations were calculated from standard curves. Oxidized glutathione (GSSG) was calculated as:

GSSG = (Total Glutathione − GSH)/2(1)

The GSSG/GSH ratio was used as an indicator of cellular oxidative stress.

### 2.4. Determination of Mitochondrial Membrane Potential (MMP) Using JC1 Dye

Changes in MMP were analyzed using the JC-1 fluorescent probe (#T3168, Thermo Fischer Scientific, Eugene, OR, USA). JC-1 exists as green-fluorescent monomers in depolarized mitochondria and forms red-fluorescent aggregates under conditions of intact mitochondrial polarization. Following completion of the respective treatments, cells from all experimental and control groups were collected, washed with PBS, and incubated with JC-1 (10 μM) for 20 min at 37 °C in the dark. Cells were subsequently washed twice by centrifugation at 600 g and subjected to flow cytometric analysis using a CytoFLEX flow cytometer (Beckman Coulter Life Sciences, Indianapolis, IN, USA). Fluorescence was detected using 525/40 BP (green) and 585/42 BP (red) filters. Mitochondrial polarization was quantified by calculating the ratio of red to green fluorescence intensity.

### 2.5. Evaluation of Mitochondrial Reactive Oxygen Species

Mitochondrial superoxide production was evaluated using MitoSOX™ Red (#M36008, Molecular Probes, Eugene, OR, USA), a mitochondria-targeted fluorogenic indicator specific to superoxide radicals. Treated and control cells were harvested, washed with PBS, and incubated with MitoSOX Red (5 μM) for 10 min at 37 °C in a humidified atmosphere containing 5% CO_2_. Following incubation, cells were washed and analyzed by flow cytometry with excitation at 488 nm and emission detection using a 585/42 BP filter. A total of 10,000 events were acquired per sample at a flow rate of 30 μL/min. Cellular debris and doublets were excluded using FSC-A versus FSC-H and FSC-A versus SSC-A gating. Histogram-based gating was applied to define the P2 population, and cells were categorized as MitoSOX-positive or -negative. Data acquisition and analysis were performed using CytExpert software (version 2.3).

### 2.6. Evaluation of Apoptosis Using Annexin V-FITC

Apoptotic cell death was assessed using an Annexin V-FITC apoptosis detection kit (#APOAF-20TST, Sigma-Aldrich, St. Louis, MO, USA) following the manufacturer’s protocol. Briefly, both control and treated cells were harvested and incubated with Annexin V-FITC (5 μL) and PI (10 μL) and then stained cells were subsequently analyzed by flow cytometry. Based on fluorescence profiles, cells were classified as viable (Annexin V^−^/PI^−^), early apoptotic (Annexin V^+^/PI^−^), or late apoptotic/necrotic (Annexin V^+^/PI^+^).

### 2.7. Total RNA Isolation and First-Strand cDNA Synthesis

Total RNA was isolated from control and treated fibroblasts samples using TRI Reagent^®^ (#T3934, Sigma-Aldrich, St. Louis, MO, USA) in accordance with the manufacturer’s guidelines. The extracted RNA was treated with RNase-free DNase I to eliminate genomic DNA contamination, followed by enzyme inactivation at 56 °C for 10 min followed by rapid cooling at 4 °C. RNA concentration and purity were assessed spectrophotometrically using a NanoDrop system by evaluating A260/A280 absorbance ratios, while RNA integrity was confirmed by visualization of intact 28S and 18S rRNA bands. Complementary DNA (cDNA) was synthesized from 200 ng of total RNA using the RevertAid first-strand cDNA synthesis kit (Thermo Fisher Scientific, Vilnius, Lithuania).

### 2.8. Quantitative Real-Time PCR Based Gene Expression Analysis

Quantitative real-time PCR (qPCR) was carried out to analyze the mRNA expression levels of glutathione peroxidase (*GPx*), superoxide dismutase (*SOD*), catalase (*CAT*), Bcl-2-associated X protein (*BAX*) and Caspase 9 (*Cas9*), using PowerUp™ SYBR^®^ Green Master Mix (#A25741, Applied Biosystems, Waltham, MA, USA). The list of primers and their details are mentioned in Supplementary File (Table S1). The amplification protocol consisted of uracil-DNA glycosylase (UDG) activation at 50 °C for 2 min, initial denaturation at 95 °C for 2 min, followed by 40 amplification cycles of denaturation at 95 °C for 15 s, gene-specific annealing for 15 s, and extension at 72 °C for 1 min. Fluorescence acquisition and threshold cycle (CT) values were obtained using the StepOnePlus™ real-time PCR system (# 272001243, Applied Biosystems, USA). PCR amplification efficiency was validated using serial template dilutions, and product specificity was confirmed through melt curve analysis to exclude non-specific amplification and primer-dimer formation.

### 2.9. Statistical Analysis

All experiments were performed using three independent biological replicates, and results are expressed as mean ± SEM. Data were analyzed by two-way ANOVA to evaluate the effects of H_2_O_2_ and melatonin treatment, followed by Tukey’s HSD post hoc test for multiple comparisons using GraphPad prism (10.1.0). Statistical significance was considered at *p* ≤ 0.05.

## 3. Results

### 3.1. H_2_O_2_-Induced Oxidative Stress in Buffalo Fibroblasts

**Figure 1:**
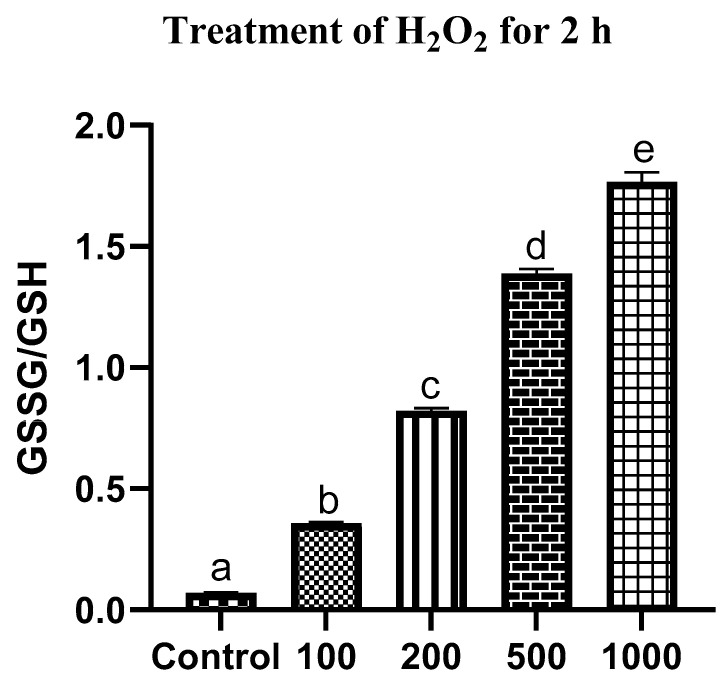
Effect of increasing concentration of H_2_O_2_ for 2 h on intracellular redox status in in vitro cultured buffalo fibroblasts. Data are presented as mean ± SEM. Different superscript letters indicate statistically significant differences (*p* ≤ 0.05).

H_2_O_2_ exposure is a commonly employed method to induce oxidative stress in cellular models. Control fibroblasts exhibited a low GSSG/GSH ratio of 0.07 ± 0.002, reflecting a well-maintained reduced intracellular environment. Following 2 h exposure to H_2_O_2_, a significant (*p* ≤ 0.05), concentration-dependent elevation in the GSSG/GSH ratio was observed (Figure 1). Cells treated with 100 and 200 μM H_2_O_2_ showed a moderate increase in the GSSG/GSH ratio to 0.35 ± 0.005 and 0.82 ± 0.008, respectively, indicating the induction of mild oxidative stress and partial redox imbalance. In contrast, exposure to higher concentrations of H_2_O_2_ (500 and 1000 μM) resulted in a significant (*p* ≤ 0.05) increase in the GSSG/GSH ratio to 1.38 ± 0.16 and 1.76 ± 0.03, respectively, demonstrating severe oxidative stress and substantial disruption of intracellular redox homeostasis.

### 3.2. Effect of H_2_O_2_ Exposure on Buffalo Fibroblast Morphology and Its Amelioration by Melatonin

**Figure 2:**
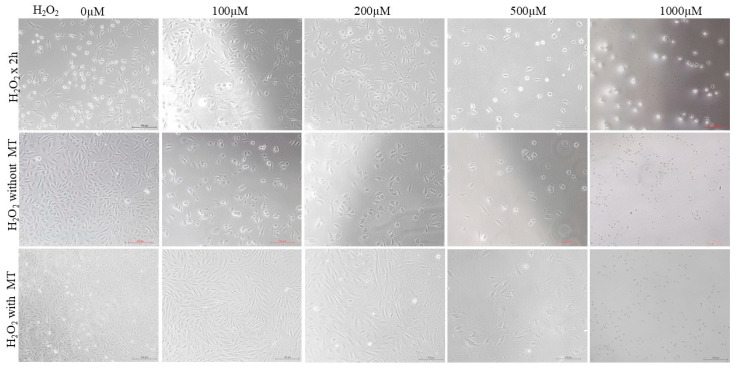
Representative images showing morphological changes in buffalo fibroblasts exposed to H_2_O_2_ (0, 100, 200, 500, and 1000 μM). Cells were exposed to H_2_O_2_ for 2 h, followed by a 72 h treatment in fresh medium either without melatonin or with melatonin (10^−9^ M). Images were captured using an inverted light microscope (Nikon Eclipse Ti, Nikon Instruments Inc., Melville, NY, USA). Scale bar = 100 μm.

Buffalo fibroblasts were exposed to increasing concentrations of H_2_O_2_ (0, 100, 200, 500, and 1000 μM) for 2 h followed by 72 h of culture with or without melatonin (10^−9^ μM). Control cells without H_2_O_2_ exhibited a typical spindle-shaped fibroblast like morphology with firm adherence and intact monolayer formation. At 100 and 200 μM H_2_O_2_, fibroblasts showed early signs of oxidative damage, characterized by cell rounding and decreased adherence, whereas higher concentrations (500 and 1000 μM) caused severe morphological disruption, extensive detachment, and a marked reduction in cell density (Figure 2). Cells subjected to increasing concentrations of H_2_O_2_ and subsequently cultured without melatonin for 72 h were largely non-viable, with the few surviving cells appearing rounded and poorly adherent, indicative of pronounced cytotoxicity. Notably, melatonin supplementation following 2 h of H_2_O_2_ exposure significantly improved cell morphology and adherence under low to moderate oxidative stress (100–200 μM), suggesting partial recovery from oxidative damage (Figure 2). However, melatonin failed to exert a protective effect at the highest H_2_O_2_ concentration (500–1000 μM), where extensive cellular damage persisted.

### 3.3. Effect of H_2_O_2_ Exposure on Mitochondrial Membrane Potential in Buffalo Fibroblasts and Its Modulation by Melatonin

**Figure 3:**
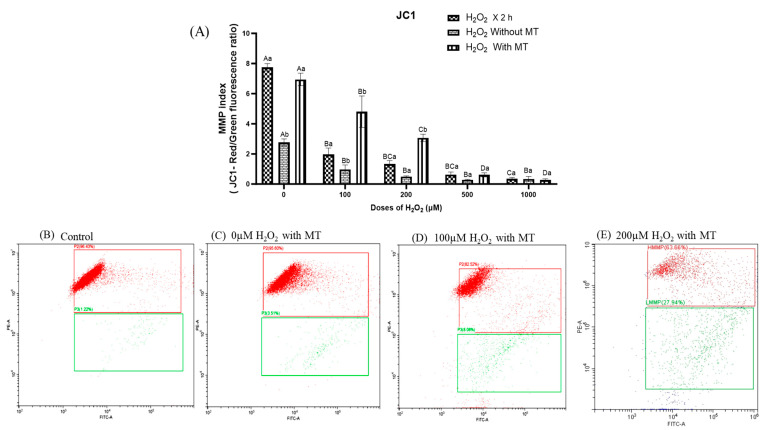
Effect of melatonin on H_2_O_2_-induced oxidative stress on mitochondrial membrane potential using JC-1 dye through flow cytometry; (**A**) Fibroblasts were exposed to H_2_O_2_ (0, 100, 200, 500, 1000 μM for 2 h) followed by 72 h treatment in fresh medium either without melatonin or with melatonin (10^−9^ M); (**B**–**E**) Dot plots show the proportion of cells with high MMP (red) and low MMP (green) at different doses. Data are presented as mean ± SEM. Different superscripts indicate significant differences (*p* ≤ 0.05); uppercase letters denote comparisons within groups, and lowercase letters denote comparisons between groups.

The MMP is a critical indicator of mitochondrial function and cellular health. In the present study, MMP in fibroblasts was assessed using the JC-1 probe, as shown in Figure 3. An increased red-to-green fluorescence ratio indicates a higher MMP, reflecting improved mitochondrial integrity. Compared to the control group, H_2_O_2_ treatment resulted in a significant (*p* ≤ 0.05), dose-dependent reduction in MMP (Figure 3A). Notably, cells maintained in culture without melatonin supplementation failed to exhibit recovery of MMP within 72 h, suggesting an inability to counteract the sustained oxidative stress. Furthermore, melatonin supplementation effectively reversed the H_2_O_2_-induced decline in MMP up to a concentration of 200 μM after 72 h (Figure 3B–E). Melatonin supplementation in cells exposed to higher H_2_O_2_ concentrations (500–1000 μM) did not exhibit any protective effect, as evidenced by decreased red to green fluorescence ration (Figure 3A), indicating irreversible mitochondrial depolarization under severe oxidative stress conditions. These findings suggest that MT plays a protective role in maintaining mitochondrial integrity under H_2_O_2_-induced oxidative stress, up to a threshold of 200 μM. Beyond this concentration, melatonin was unable to preserve MMP.

### 3.4. Melatonin-Mediated Modulation of ROS Production in Buffalo Fibroblasts Following H_2_O_2_ Exposure

**Figure 4:**
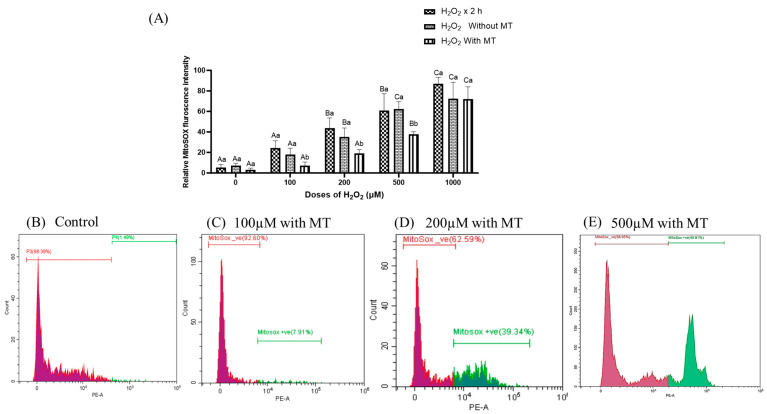
(**A**) Effect of melatonin on mitochondrial ROS production in buffalo fibroblasts exposed with different concentration of H_2_O_2_ assessed by MitoSox Red dye using flow cytometry; (**B**–**E**) Histogram showing MitoSOX positive and MitoSOX negative in H_2_O_2_ (0, 100, 200, and 500 μM) treated cells, followed by melatonin supplementation (10^−9^ M) for 72 h. Data are presented as mean ± SEM. Different superscripts indicate significant differences (*p* ≤ 0.05); uppercase letters denote comparisons within groups, and lowercase letters denote comparisons between groups.

To investigate the effect of melatonin on mitochondrial ROS production in H_2_O_2_-induced oxidative stress in fibroblasts, MitoSOX Red-a mitochondrial-targeted superoxide indicator capable of penetrating cells and selectively localizing to mitochondria was employed. Exposure of H_2_O_2_ resulted in a significant, dose-dependent increase in mitochondrial ROS levels at 2 h (Figure 4A). Notably, ROS levels did not recover even when cells were maintained in culture for 72 h across all doses (Figure 4A). However, melatonin supplementation (10^−9^ M) for 72 h significantly (*p* ≤ 0.05) reduced ROS production, indicating its potent mitochondrial antioxidant activity compared to cells cultured without melatonin, with a protective effect observed up to 500 μM H_2_O_2_ after 2 h of exposure (Figure 4B–E).

**Figure 5:**
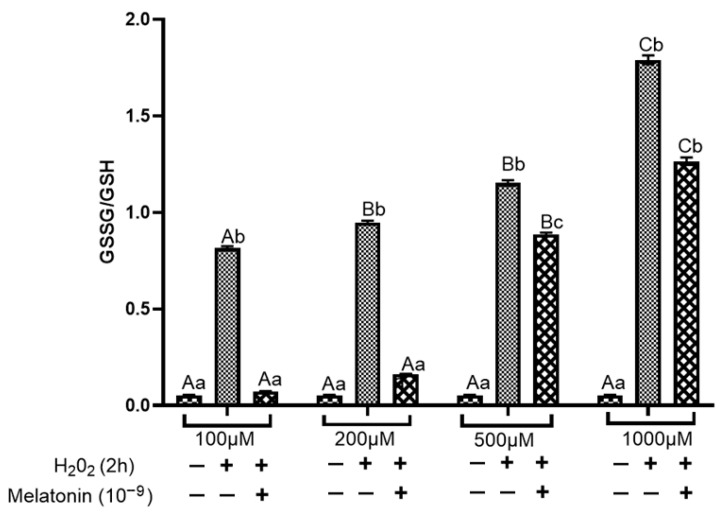
Effect of H_2_O_2_-induced oxidative stress and melatonin treatment on the GSSG/GSH ratio in buffalo fibroblasts. The GSSG/GSH ratio serves as a key indicator of intracellular redox status and oxidative stress. Data are presented as mean ± SEM. Different superscripts indicate significant differences (*p* < 0.05); uppercase letters denote comparisons within groups, and lowercase letters denote comparisons between groups.

Oxidative stress in cultured buffalo fibroblasts was further evaluated by determining the ratio of oxidized to reduced glutathione (GSSG/GSH). Control cells at 72 h of culture exhibited a low GSSG/GSH ratio, indicative of a predominantly reduced intracellular environment. In contrast, exposure to H_2_O_2_ for 2 h led to a significant (*p* ≤ 0.05), concentration-dependent increase in the GSSG/GSH ratio, reflecting severe oxidative stress and disruption of cellular redox homeostasis at 72 h (Figure 5). Melatonin supplementation significantly (*p* ≤ 0.05) attenuated this increase in cells treated with low to moderate concentrations of H_2_O_2_ (100–200 μM), thereby partially restoring redox balance. Although melatonin reduced the GSSG/GSH ratio at concentrations of 500 μM H_2_O_2_, the cells continued to exhibit signs of oxidative stress. At higher H_2_O_2_ concentrations, melatonin failed to normalize the GSSG/GSH ratio, suggesting that excessive oxidative stress overwhelms the endogenous antioxidant defence system.

### 3.5. Dose-Dependent Impact of H_2_O_2_ on Fibroblast Viability and Apoptosis and Its Attenuation by Melatonin Under Oxidative Stress

**Figure 6:**
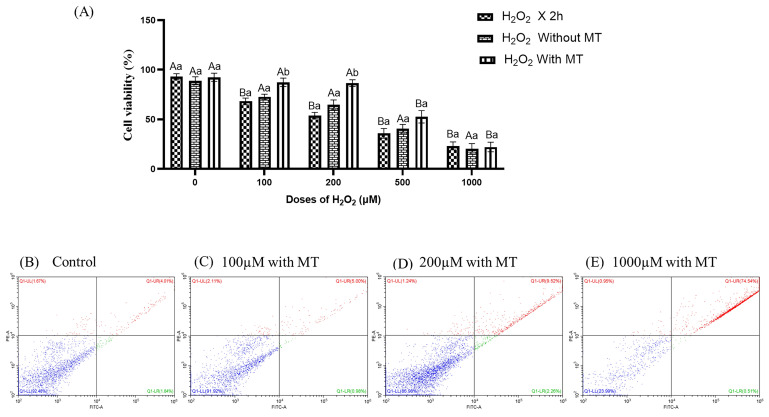
Effect of melatonin on H_2_O_2_-induced apoptosis in buffalo fibroblasts assessed by Annexin V-FITC flow cytometry. (**A**) Fibroblasts were exposed to H_2_O_2_ (0, 100, 200, 500, 1000 μM; 2 h) followed by 72 h in fresh medium with or without melatonin (10^−9^ M); (**B**–**E**) Dot plots show the proportion of viable, early apoptotic, late apoptotic, and necrotic cells at different doses. Data are presented as mean ± SEM. Different superscripts indicate significant differences (*p* ≤ 0.05); uppercase letters denote within-group comparisons, and lowercase letters denote between-group comparisons.

Annexin V-FITC/PI-based flow cytometric analysis was employed to quantify apoptosis and cell death in buffalo fibroblasts subjected to H_2_O_2_-induced oxidative stress and to evaluate the cytoprotective potential of melatonin. Exposure to increasing concentrations of H_2_O_2_ resulted in a dose-dependent elevation in the proportion of apoptotic and dead cells, confirming the induction of oxidative stress-mediated cellular damage (Figure 6A). Notably, melatonin supplementation significantly attenuated cell death at 72 h of culture (*p* ≤ 0.05) in fibroblasts exposed to H_2_O_2_ concentrations up to 200 µM, as compared to cells cultured without melatonin (Figure 6B–E). However, at H_2_O_2_ concentrations exceeding 200 µM, melatonin failed to confer significant cyto-protection, suggesting that its antioxidative and anti-apoptotic efficacy is concentration-dependent and becomes insufficient under severe oxidative stress. These findings indicate that melatonin exerts a potent anti-apoptotic effect within a defined oxidative stress threshold, beyond which irreversible cellular damage may prevail.

### 3.6. Effect of Melatonin on Antioxidant Enzymes (GPx, SOD, CAT) and Apoptotic Markers (BAX, Caspase 9) Under H_2_O_2_-Induced Oxidative Stress

**Figure 7:**
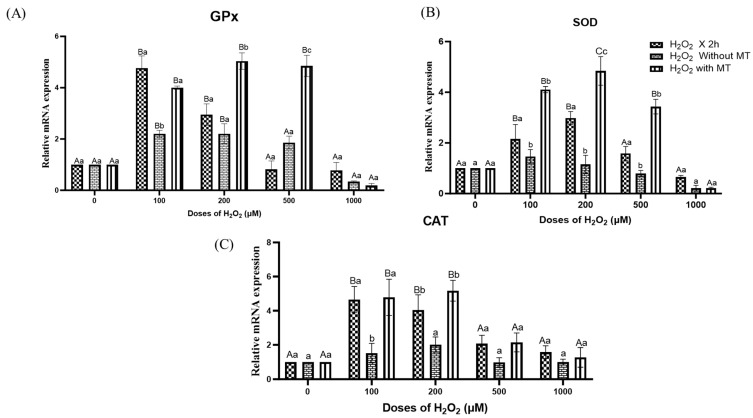
Effect of melatonin on H_2_O_2_ induced changes in antioxidant gene expression, glutathione peroxidase (GPx) (**A**), superoxide dismutase (SOD) (**B**), and catalase (CAT) (**C**) in buffalo fibroblasts. Fibroblasts were exposed to H_2_O_2_ (0, 100, 200, 500, 1000 μM; 2 h) followed by 72 h in fresh medium with or without melatonin (10^−9^ M). Data are presented as mean ± SEM (*n* = 3). Different superscripts indicate significant differences (*p* < 0.05); uppercase letters denote within-group comparisons, and lowercase letters denote between-group comparisons.

The expression of key antioxidant enzymes, glutathione peroxidase (GPx) (Figure 7A), superoxide dismutase (SOD) (Figure 7B), and catalase (CAT) (Figure 7C), was evaluated in buffalo fibroblasts exposed to increasing concentrations of H_2_O_2_, followed by culture for 72 h in the presence or absence of melatonin. Short-term exposure to H_2_O_2_ (2 h) alone resulted in a dose-dependent modulation of GPx, SOD and CAT transcript levels (Figure 7). At moderate oxidative stress levels (100–200 µM), a significant (*p* ≤ 0.05) upregulation of all three antioxidant enzymes was observed in melatonin treated group compared to the untreated controls, indicating an adaptive cellular antioxidant response. Interestingly, at 500 µM H_2_O_2_, GPx and SOD were significantly upregulated (Figure 7A,B), whereas CAT expression (Figure 7C) remained unchanged, suggesting a selective activation of enzymatic defences. In contrast, exposure to higher H_2_O_2_ concentrations (>500 µM) led to a pronounced reduction in GPx, SOD, and CAT transcript levels, suggesting exhaustion of the cellular redox buffering capacity and impaired antioxidant defence. Collectively, these results demonstrate that melatonin sustains antioxidant enzyme expression under moderate oxidative stress and selectively modulates GPx and SOD at intermediate stress levels, but its protective effect diminishes under severe oxidative insult.

**Figure 8:**
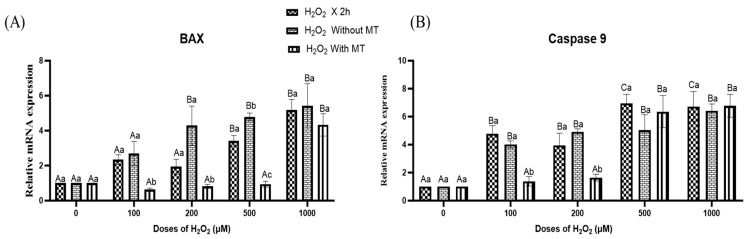
Effect of melatonin on H_2_O_2_-induced changes in apoptotic gene expression, BAX (**A**) and Caspase 9 (**B**) in buffalo fibroblasts. Fibroblasts were exposed to H_2_O_2_ (0, 100, 200, 500, 1000 μM; 2 h) followed by 72 h in fresh medium with or without melatonin (10^−9^ M). Data are presented as mean ± SEM (*n* = 3). Different superscripts indicate significant differences (*p* < 0.05); uppercase letters denote within-group comparisons, and lowercase letters denote between-group comparisons.

In addition to antioxidant profiling, the expression of the apoptotic markers BAX (Figure 8A) and Caspase 9 (Figure 8B) was assessed in buffalo fibroblasts exposed to increasing concentrations of H_2_O_2_, followed by 72 h of culture in the presence or absence of melatonin. The oxidative stress (2 h H_2_O_2_ exposure) resulted in a significant (*p* ≤ 0.05) upregulation of BAX and Caspase 9 transcripts, indicating early activation of apoptotic signalling (Figure 8). Notably, these elevated transcript levels persisted after 72 h of culture in untreated cells, suggesting sustained pro-apoptotic stress. In contrast, fibroblasts cultured with melatonin (10^−9^ M) for 72 h exhibited a reduction in BAX and Caspase 9 expression, particularly at H_2_O_2_ concentrations up to 200 µM (Figure 8), underscoring the protective role of melatonin in attenuating apoptosis under moderate oxidative stress. Collectively, these findings indicate that melatonin alleviates H_2_O_2_-induced apoptotic signalling by suppressing the transcriptional activation of key apoptotic mediators.

## 4. Discussion

In the present study, we established an adaptive tolerance model to H_2_O_2_ in buffalo fibroblasts, wherein exposure to a sublethal dose of H_2_O_2_ enhanced the cellular antioxidant capacity. However, higher doses failed to compensate for the induced oxidative stress, resulting in loss of cyto-protection. Furthermore, our findings reveal that melatonin-mediated stimulation of glutathione synthesis underlies its cytoprotective effects in a threshold-dependent manner, delineating a critical redox window beyond which oxidative damage becomes irreversible. The intracellular glutathione redox system constitutes a central antioxidant defence mechanism and serves as a sensitive indicator of cellular redox status [26]. In this study, H_2_O_2_ exposure resulted in a significant increase in the GSSG/GSH ratio, reflecting a shift toward an oxidized intracellular environment. Such redox imbalance is a hallmark of oxidative stress and has been widely associated with impaired antioxidant capacity, metabolic dysfunction, and reduced cell survival in fibroblasts and similar findings were observed in other somatic cell types [27,28]. The increase in the GSSG/GSH ratio indicates depletion of reduced glutathione reserves and compromised detoxification of peroxides, thereby exacerbating oxidative injury [23].

Melatonin supplementation markedly reduced the GSSG/GSH ratio under mild to moderate oxidative stress conditions, representing its ability to restore intracellular redox homeostasis. This protective effect can be credited to melatonin’s dual antioxidant action, the direct scavenging of ROS, including hydrogen peroxide and hydroxyl radicals, and indirect regulation of glutathione metabolism through stimulation of glutathione synthesis and regeneration [29,30]. Similar to our findings, reference [23] also observed that melatonin failed to normalize the redox imbalance at higher H_2_O_2_ concentrations, suggesting that excessive oxidative burden overpowers the endogenous and melatonin-supported antioxidant defences, leading to irreversible cellular damage.

Mitochondria are recognized as a primary source of ROS generation [31]. While ROS function as essential signalling molecules under physiological conditions, their excessive accumulation under pathological states leads to oxidative stress and cellular dysfunction [32]. The MMP is a key determinant of mitochondrial health, as it sustains respiratory chain activity, drives adenosine triphosphate (ATP) synthesis, and preserves normal mitochondrial function. Loss of MMP disrupts cellular energy homeostasis and ultimately triggers cell death [33,34]. Melatonin treatment effectively preserved MMP in fibroblasts exposed to mild and moderate oxidative stress, indicating stabilization of mitochondrial integrity. These findings are in agreement with previous studies demonstrating that melatonin preferentially accumulates within mitochondria, improves electron transport chain efficiency, reduces electron leakage, and preserves mitochondrial bioenergetics [35,36]. Consistent with mitochondrial dysfunction, H_2_O_2_ exposure significantly increased mitochondrial ROS production, as demonstrated by increased MitoSOX fluorescence. Excessive mitochondrial ROS amplifies lipid peroxidation, protein oxidation, and mitochondrial DNA damage, promoting activation of intrinsic apoptotic pathways [37]. Melatonin significantly reduced mitochondrial ROS levels under mild to moderate oxidative stress, confirming its role as a potent mitochondria-targeted antioxidant [13]. Morvaridzadeh et al. [18] performed a meta-analysis demonstrating melatonin supplementation remarkable effects on increased level of total antioxidant capacity (TAC), GSH levels, SOD, GPx, glutathione reductase activities, and a significant decrease in serum levels of malondialdehyde (MDA). In the present work, melatonin displayed antioxidant actions by stimulating the synthesis of GPx and SOD, as well as by promoting its enzymatic recycling in cells to ensure it remained primarily in its reduced form. Similar melatonin-mediated induction of antioxidant enzymes has been reported in various in vitro, in vivo, and cryobiological models [20,38,39]. In contrast, the decline in GPx and SOD expression at higher H_2_O_2_ concentrations suggests exhaustion of antioxidant defence mechanisms, further stressing the limitations of cellular repair processes under extreme oxidative stress [22].

At the molecular level, oxidative stress induced by H_2_O_2_ was associated with activation of apoptotic signalling pathways, as indicated by increased apoptotic cell populations and altered expression of apoptosis-related genes. These findings reflect the initiation of programmed cell death in response to excessive oxidative damage. Melatonin supplementation significantly improved cell viability and reduced apoptosis under lower oxidative stress conditions, which was associated with decreased expression of apoptotic markers. These observations are consistent with previous reports demonstrating that melatonin suppresses apoptosis by modulating mitochondrial signalling, inhibiting caspase activation, and regulating the balance between pro- and anti-apoptotic proteins [40,41].

## 5. Conclusions

This study delineates a critical redox threshold that determines the balance between adaptive survival and irreversible injury in buffalo fibroblasts under oxidative stress. Sublethal H_2_O_2_ exposure induced a protective adaptive response marked by enhanced antioxidant capacity, whereas higher oxidative loads disrupted glutathione redox homeostasis, impaired mitochondrial function, elevated mitochondrial ROS, and activated apoptotic pathways. Notably, melatonin conferred significant cyto-protection only within a defined window of oxidative stress by restoring GSH/GSSG balance, preserving MMP, suppressing mitochondrial ROS accumulation, and upregulating key antioxidant enzymes, thereby improving cell viability and proliferative potential. However, this protective effect was lost under severe oxidative conditions, emphasizing the biological limitation of antioxidant-mediated rescue once cellular damage becomes irreversible. Collectively, these findings establish a robust in vitro model of oxidative adaptation and define the mechanistic boundaries of melatonin-mediated protection, offering valuable insights for optimizing fibroblast culture systems in applications such as genome editing, SCNT, and long-term in vitro maintenance of somatic cells for livestock biotechnology.

## Figures and Tables

**Table 1:** Experimental design for H_2_O_2_-induced oxidative stress and melatonin supplementation in buffalo fibroblasts.

Group	H_2_O_2_ (2 h)	72 h Culture	Melatonin, 10^−9^ M (MT)
Control	–	–	–
H_2_O_2_ exposure for 2 h only	+	–	–
H_2_O_2_ exposure followed by 72 h culture	+	+	–
H_2_O_2_ exposure followed by 72 h culture with MT	+	+	+

## Data Availability

The data supporting the findings of this study are available within the article and its Supplementary Materials.

## Supplementary Materials

The following supporting information can be downloaded at: https://www.mdpi.com/article/10.3390/antiox15040508/s1, Figure S1: Representative figure of characterization of buffalo fibroblasts. Positive signals for vimentin and no signals for cytokeratin-18 indicate that cultured cells belonged to fibroblast cell type. Tubulin staining was used as positive staining control. Table S1: List of primer sequence and amplicon length used for qPCR.

## Abbreviations

CAT	Catalase
GPx	Glutathione peroxidase
GR	Glutathione reductase
GSH	Reduced glutathione
GSSG	Oxidized glutathione (Glutathione Disulfide)
H_2_O_2_	Hydrogen peroxide
MMP	Mitochondrial membrane potential
MDA	Malondialdehyde
MT	Melatonin
qPCR	Real-time polymerase chain reaction
ROS	Reactive oxygen species
SCNT	Somatic cell nuclear transfer
SOD	Superoxide dismutase
TAC	Total antioxidant capacity
