# Supplementary material for: Melatonin Attenuates H2O2-Induced Oxidative Stress by Restoring Redox Balance, Mitochondrial Integrity and Reducing Apoptosis in Buffalo Fibroblasts
Source: Antioxidants (Basel). 2026 Apr 20;15(4):508. doi: 10.3390/antiox15040508 (PMC13113247; doi:10.3390/antiox15040508)
Supplement: Supplementary file 1 [file antioxidants-15-00508-s001.zip › antioxidants-4153064-supplementary.pdf]

Melatonin Attenuates H_2O_2-Induced Oxidative Stress by Restoring Redox Balance, Mitochondrial Integrity and Reducing Apoptosis in Buffalo Fibroblasts

Priya Dahiya, Manu Mangal, Srishti Bhatia, Neha Sharma, Ashish Sindhu, Bhavya Maggo, Meeti Punetha*, Renu Bala, Pradeep Kumar, Dharmendra Kumar*

Animal Physiology and Reproduction Division, ICAR-Central Institute for Research on Buffaloes, Sirsa Road, Hisar, India-125001

*Authors for correspondence: dkumarbt@gmail.com; meetipunetha283@gmail.com

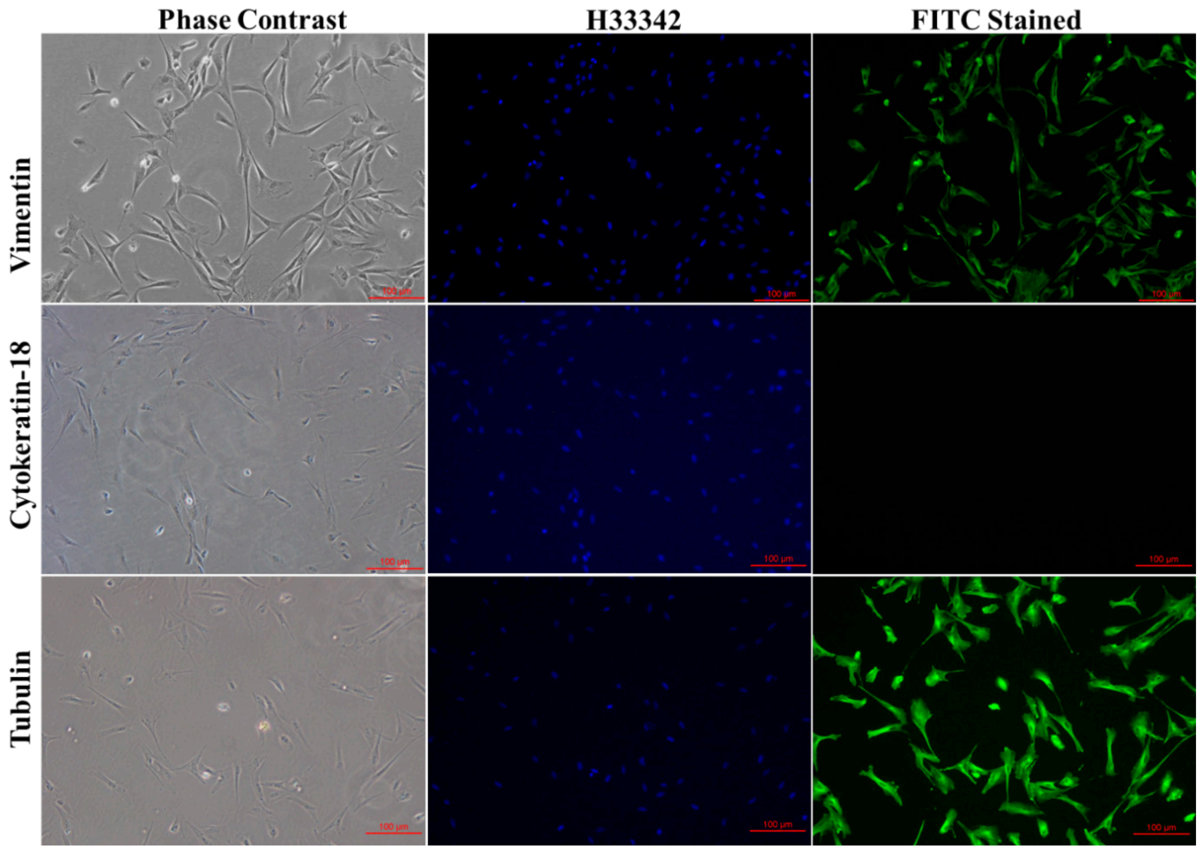

Figure S1: Representative figure of characterization of buffalo fibroblasts. Positive signals for vimentin and no signals for cytokeratin-18 indicate that cultured cells belonged to fibroblast cell type. Tubulin staining was used as positive staining controls

Table S1: List of primer sequence and amplicon length used for qPCR.

**Gene**	**Nucleotide Sequences (5’-3’)**	**Amplicon length (bp)**	**Annealing temperature (^{o}C)**	**Accession number**
GPx	F: ACGCCACTGCTCTAATGACC<br>R: GGACAGCAGGGTTTCAATGT	176	58	>XM_006053253.3
SOD	F: TGGAGAAGGGTGATGTTACAG<br>R: TTAGGGCTCAGATTTGTCCA	106	58	>XM_025294324.2
CAT	F: AACCCTCAAACGCACCTGAA<br>R: ATGTGCCTGTGTCCATCTGG	125	60	>XM_044929272.2
Bax	F: AGGGTTTCATCCAGGATCGA<br>R: AAGTCCAATGTCCAGCCCAT	346	60	>XM_006050927.4
Caspase9	F: TGAAACAGCATTAGCGACCC<br>R: GTGTTCTACTCCACCTTCCC	150	58	>XM_006072791.4
GAPDH	F: ACCCAGAAGACTGTGGATGG<br>R: ATGCCTGCTTCACCACCTTC	247	58	>XM_006065800.4
